# Optically Stimulated Nanodosimeters with High Storage Capacity

**DOI:** 10.3390/nano9081127

**Published:** 2019-08-05

**Authors:** David Van der Heggen, Daniel R. Cooper, Madeleine Tesson, Jonas J. Joos, Jan Seuntjens, John A. Capobianco, Philippe F. Smet

**Affiliations:** 1 LumiLab, Department of Solid State Sciences, Ghent University, Krijgslaan 281-S1, 9000 Gent, Belgium; 2 Medical Physics Unit, McGill University, Cedars Cancer Centre, 1001 Décarie Blvd, Montreal, QC H4A 3J1, Canada; 3 Department of Chemistry and Biochemistry and Centre for NanoScience Research, Concordia University, 7141 Sherbrooke St. W., Montreal, QC H4B 1R6, Canada

**Keywords:** persistent phosphors, dosimetry, optically stimulated luminescence, nanophosphor, thermoluminescence

## Abstract

In this work we report on the thermoluminescence (TL) and optically stimulated luminescence (OSL) properties of β-Na(Gd,Lu)F_4_:Tb^3+^ nanophosphors prepared via a standard high-temperature coprecipitation route. Irradiating this phosphor with X-rays not only produces radioluminescence but also leads to a bright green afterglow that is detectable up to hours after excitation has stopped. The storage capacity of the phosphor was found to be (2.83 ± 0.05) × 10^16^ photons/gram, which is extraordinarily high for nano-sized particles and comparable to the benchmark bulk phosphor SrAl_2_O_4_:Eu^2+^,Dy^3+^. By combining TL with OSL, we show that the relatively shallow traps, which dominate the TL glow curves and are responsible for the bright afterglow, can also be emptied optically using 808 or 980 nm infrared light while the deeper traps can only be emptied thermally. This OSL at therapeutically relevant radiation doses is of high interest to the medical dosimetry community, and is demonstrated here in uniform, solution-processable nanocrystals.

## 1. Introduction

Luminescent materials, or phosphors, possess the ability to absorb high-energy radiation and convert it into light with a typically lower energy. These materials are widely applied as color converters in lighting applications [1,2,3] or as scintillators to detect high-energy radiation [4,5,6,7]. Usually the absorption of high-energy radiation is immediately followed by the emission of light. Depending on the type of excitation, the process is called photo- or radioluminescence. However, some phosphors can store part of the energy that is provided to them during excitation and when this energy is released, it can give rise to emission long after the excitation has stopped, at times that are considerably longer than the photo- or radioluminescence lifetimes [8]. These materials cover a large spectral range, featuring emission from the Ultraviolet C (UVC) range which can be used for sterilization or disinfection [9] up to the red and near-infrared range, enabling the use of persistent phosphors in in vivo medical imaging [10,11,12]. Depending on the envisioned application, the storage or afterglow properties of the materials can be tuned by adding co-dopants [13,14] or by slightly changing the host composition [15].

If the energy storage is stable at room temperature the material is termed a storage phosphor [16,17]. These materials are ideal for radiation dosimetric purposes but are also used as imaging plates in medical applications [18,19]. Storage phosphors, such as BaFBr:Eu^2+^ or the well-known LiF:Mg,Ti, require an external stimulus, such as heat or light, to release the stored energy [17,20], but in some cases the thermal energy present at room temperature is sufficient to release the stored energy and the phosphors spontaneously emit light. This emission is called afterglow and hence these luminescent materials are also named afterglow phosphors [8]. The green-emitting SrAl_2_O_4_:Eu,Dy is one of the best known afterglow phosphors and it is widely applied in, for example, safety signage and watch dials. Since its discovery by Matsuzawa et al. [21], many more of these long afterglow phosphors have been discovered, though most of them are Eu^2+^-activated oxides [22]. Persistent phosphors with a particle size in the nanoscale have been synthesized by the size reduction of micron-sized phosphors [23,24,25] or by bottom-up methods, as in the case of ZnGa_2_O_4_:Cr^3+^ [26]. It remains a challenge to achieve a high storage capacity in nano-sized phosphors, as often thermal annealing is required, but specific methods have been developed to allow this thermal annealing while avoiding grain growth [27].

A recent systematic study of the radioluminescence properties of nanoparticles with the general composition β-NaLnF_4_ revealed that β-Na(Gd,Lu)F_4_:Tb^3+^ exhibits a strong afterglow after exposure to X-rays [28]. The isostructural NaYF_4_:Er [29] and NaGdF_4_:Yb,Er [30] have already been suggested for medical imaging applications based on their up-conversion properties, but the strong afterglow and stable charge storage in β-Na(Gd,Lu)F_4_:Tb^3+^ offer new possibilities to use this type of nanoparticle in medical and personal dosimetric applications. In the following, we discuss the thermoluminescence (TL) and optically stimulated luminescence (OSL) of this phosphor and we show that this material differs from conventional persistent phosphors because it possesses an exceptionally high storage capacity which, based on performance, places it among the commercially available afterglow phosphors but with the added advantage that it is nano-sized with a well-defined size distribution. This opens up applications for nano-sized dosimeters that could be used to quantify radiation dose in cellular structures or could be applied in larger concentrations as highly sensitive dosimeters applicable in radiation therapy and radiation protection.

## 2. Materials and Methods

All nanoparticle synthesis reagents were purchased from Sigma-Aldrich (Oakville, ON, Canada). Lanthanide salts were purchased at the highest available purity (≥99.99% for Lu(III) chloride hexahydrate; 99.999% for Gd(III) chloride hexahydrate and Tb(III) chloride hexahydrate). Other reagents/solvents consisted of sodium hydroxide (pellets, semiconductor grade, 99.99% trace metals basis), ammonium fluoride (≥99.99% trace metals basis), hexane (mixture of isomers, ≥98.5%), and technical grade (90%) 1-octadecene and oleic acid. Nanoparticles were synthesized in a manner similar to that previously described [28], with reagent quantities scaled up by 50%—the initial solution consisted of 1.5 mmol total of hydrated lanthanide chlorides dissolved in 22.5 mL 1-octadecene and 9 mL oleic acid in a 100 mL round-bottom flask, and the methanol-based precursor solution consisted of 15 mL containing 3.75 mmol NaOH and 6 mmol NH_4_F. During the post-synthesis washing, 60 μL of nanoparticle suspension was diluted in 2 mL hexanes for dynamic light scattering (DLS) measurements and preparation of transmission electron microscopy (TEM) samples. One aliquot of ~35 mg was dried overnight under vacuum for powder X-ray diffraction (XRD) measurements. The remaining pelleted nanoparticles were stored under a small volume of ethanol until use.

The crystal phase of the nanoparticles was evaluated by XRD using a Scintag XDS-2000 diffractometer (Thermo Scientific; Waltham, MA, USA) equipped with a Si(Li) Peltier-cooled solid state detector and Cu Kα source (*λ* = 1.540562 Å) operating at a generator power of 45 kV and 40 mA. The 2θ scan range was set from 10–90° with a step size of 0.02° and a dwell time of 0.5 s. Quartz zero background insert disks were used to support the nanoparticle powder. Nanoparticle sizes and morphologies were studied by TEM. TEM images were obtained using a JEOL JSM2100F TEM (Akishima; Tokyo, Japan) operating at 200 kV. Samples were prepared by depositing 10 μL droplets of nanoparticles in hexanes on 300 mesh Carbon Type-B Formvar film-coated Cu grids (Ted Pella; Redding, CA, USA). Nanoparticle dimensions were determined by manual measurements using ImageJ (*n* = 150). DLS measurements and analysis were performed with a Malvern Zetasizer Nano ZSP and Zetasizer software (Malvern Panalytical Ltd.; Malvern UK). Samples for inductively coupled plasma mass spectrometry (ICP-MS) were prepared by dissolving ~1 mg of nanoparticle powder in concentrated HCl overnight at 115 °C then diluting to the appropriate concentration with 5% aqueous HNO_3_. The measurements were conducted using an Agilent 7500ce ICP-MS instrument (Agilent; Santa Clara, Ca, USA) equipped with a quartz Scott-type spray chamber, off-axis Omega lens ion focusing, and an octopole reaction cell with a quadrupole mass spectrometer analyzer with 4 mL/min helium. Z_eff_ calculations were performed with Auto-Z_eff_ software [31].

Thermoluminescence glow curves were measured in a home-built setup inside a Siemens D5000 X-ray diffractometer (Cu anode, not filtered, operated at 40 kV, 40 mA) yielding an estimated air kerma rate of 15 Gy min^−1^ at the position of the sample. A lower dose rate was achieved using an aluminum filter with a thickness of 3 mm. Based on the atomic composition of the nanoparticles, the change in dosimetric response due to spectral changes caused by the 3 mm Al filter is not expected to exceed 5% in this energy region; therefore, using 3 mm filtration is not expected to affect the linearity test at the low dose rates reported in this work. The heating was performed by a resistive heating element at a rate of 1 °C/s, with temperature feedback using a USB-6002 DAQ device (National Instruments; Austin, TX, USA). The emission spectra were measured by means of a QE65000 spectrometer (Ocean Optics; Largo, FL, USA). The emission intensity was determined with an ILT 1700 calibrated photometer (International Light Technologies; Peabody, MA, USA) equipped with a photopic filter (YPM). The absolute trapping capacity was determined using the procedure described in a previous work [32]. Optical stimulation was performed by irradiation with an 808 nm (RLDB808-500-5, 500mW, Roithner Lasertechnik; Vienna, Austria) or a 980 nm (RLDH980-200-3, 200mW, Roithner Lasertechnik; Vienna, Austria) infrared laser while monitoring the light emission with the ILT 1700 photometer at low doses and the QE65000 spectrometer at higher doses. A shortpass filter (650 nm cut-off, OD4, Edmund Optics; Barrington, NJ, USA) was used to discriminate the Tb^3+^ emission from reflected infrared light. The OSL measurements and the measurement of the storage capacity were performed on a polymer disk with a diameter of 18 mm and a thickness of 0.9 mm, containing 4 mg of Na(Lu_0.65_Gd_0.2_Tb_0.15_)F_4_ which was suspended in 255 mg of PDMS (Sylgard 184). To avoid the degradation of the polymer and to ensure good thermal conductivity while heating, the thermoluminescence measurements were performed on bare powder. Pictures for the imaging experiment were taken with a Nikon D3200 camera (Nikon Corporation; Tokio, Japan) equipped with a bandpass filter (520 nm center wavelength, 70 nm bandwidth, OD6, Edmund Optics; Barrington, NJ, USA) and with an integration time of 20 s. A copper plate was used as a mask to pattern the layer during irradiation. 

## 3. Results and Discussion

**Figure 1:**
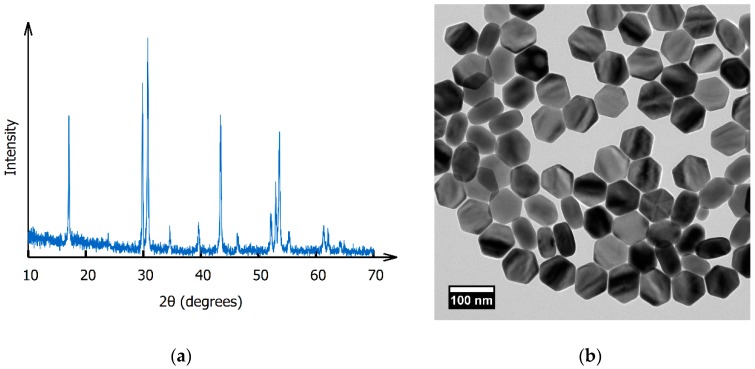
(**a**) XRD pattern of the β-Na(Gd,Lu)F_4_:Tb^3+^ nanophosphor; (**b**) TEM image of β-Na(Gd,Lu)F_4_:Tb^3+^ nanophosphors. Note that a portion are oriented on their sides.

TEM images revealed nanocrystals with irregular hexagonal prism morphology, with long diagonals of 87.0 ± 4.9 nm (coefficient of variation 5.6%), as shown in Figure 1. DLS measurements of oleate-capped nanocrystals dispersed at ~1 mg/mL in hexanes gave a *Z-ave* solvodynamic diameter of 85.52 ± 0.27 nm (PdI = 0.063 ± 0.014, indicative of a uniform colloid). Based on the XRD pattern (Figure 1), the nanocrystals were assigned to the hexagonal phase by comparison to JCPDS cards 27-0699 (β-NaGdF_4_) and 27-0726 (β-NaLuF_4_) [28]. ICP-MS revealed the actual ratio of Gd:Tb:Lu to be 19.95:15.47:64.58, in good agreement with the nominal composition of 20:15:65. This composition was chosen based on the results of Zhang et al. [33], which showed optimal radioluminescence intensity in NaGdF_4_ for a Tb^3+^ concentration of 15%.

**Figure 2:**
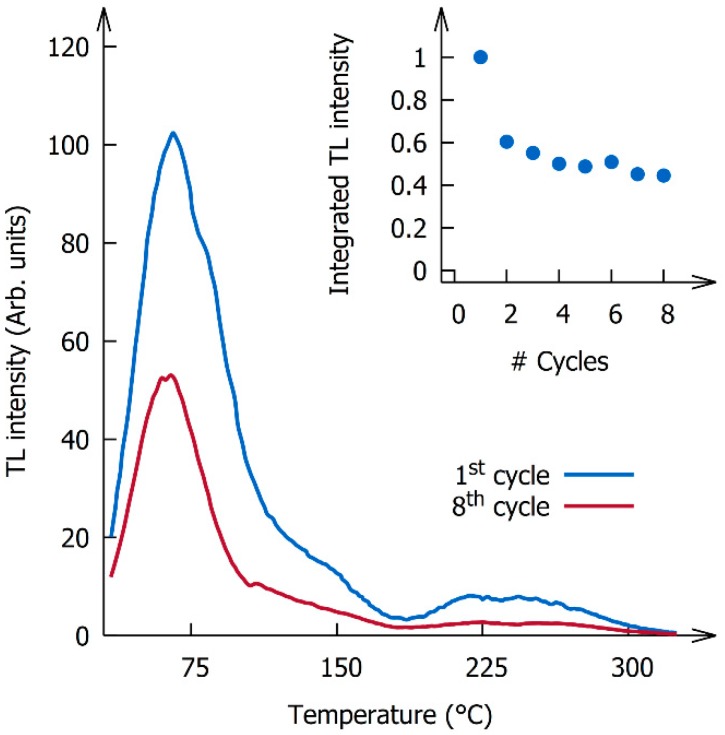
Thermoluminescence glow curves recorded after 600 s of X-ray irradiation (~150 Gy). The inset shows the total integrated thermoluminescence intensity as a function of the number of charging–heating cycles.

**Figure 3:**
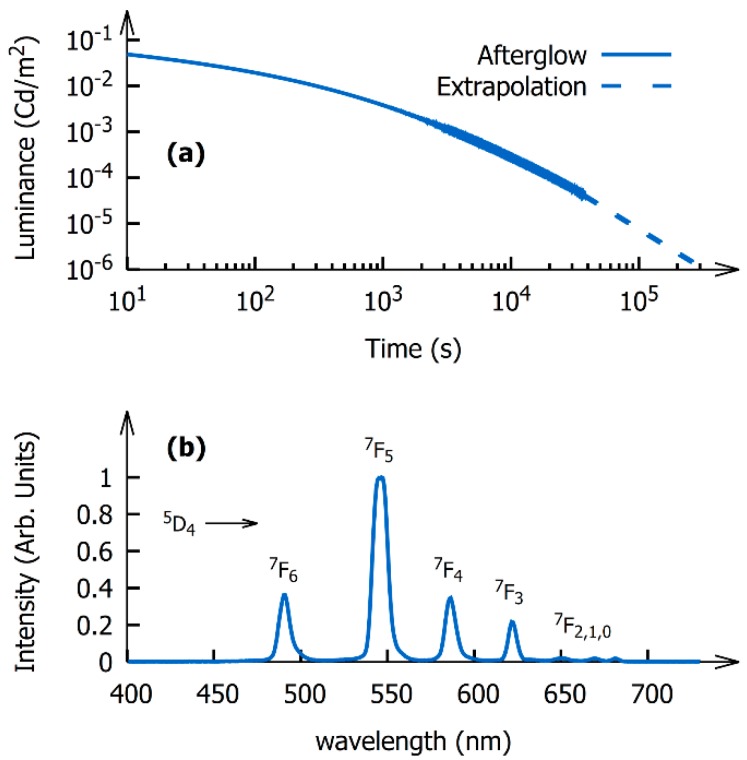
(**a**) Afterglow curve of the polymer layer containing the Na(Gd,Lu)F_4_:Tb^3+^ nanoparticles; (**b**) normalized radioluminescence spectrum of the Na(Gd,Lu)F_4_:Tb^3+^ nanoparticles.

The thermoluminescence glow curves recorded after irradiating the sample with X-rays for 600 s (~150 Gy) are shown in Figure 2. The glow curve is dominated by a peak at 65 °C but three additional peaks can be identified at higher temperatures around 130, 220, and 260 °C. This is in line with the results reported by Krumpel et al. for Tb^3+^- and Gd^3+^-doped NaLaF_4_ [34]. Dose dependence measurements indicated that there was no significant shift in the glow peak positions with increasing dose, suggesting that the trapping process is well described by first-order kinetics. The thermoluminescence spectra remained unchanged over the entire temperature range and are identical to the radioluminescence spectrum shown in Figure 3b. The spectrum consists of the characteristic ^5^D_4_ → ^7^F_J_ emission lines of Tb^3+^ and exhibits no other features. No emission from the ^5^D_3_ level was observed, which is not surprising given the high doping concentration, enabling fast cross relaxation [35]. 

The inset in Figure 2 shows that the total thermoluminescence intensity decreases with each successive charging and heating cycle, especially during the first cycles. Even though the total intensity changes, the shape of the glow curve appears to remain unaffected by the degradation. Simultaneous with the decrease in intensity, the powder changes color and turns brownish. This change in color appears to be irreversible, indicating that the discoloration is not a photochromic effect related to deep trapping centers, but is presumably related to oleate oxidation during the high temperature read-out step (up to 300 °C) when recording the thermoluminescence glow curves. 

To quantify how much energy can be stored in the material, the storage capacity of the phosphor was determined. This was done by integrating the afterglow of a polymer layer (shown in Figure 3a) containing a known amount of powder (1.60 ± 0.05 mg/cm^2^) according to the procedure that was introduced and validated in a previous work [32]. The storage capacity is defined as the number of photons emitted per gram of phosphor. It allows for comparison of the performance of different persistent phosphors in an absolute way because it is independent of the phosphor’s composition, its emission spectrum, and the sample geometry, in stark contrast to decay curves which are usually reported and are generally measured on infinitely thick powder samples or ceramics and expressed in spectrally dependent units such as Cd/m^2^ or mW/m^2^/sr.

With an average photon energy of 2.22 eV and a luminous efficacy of 504 lm/W, the storage capacity of the phosphor was found to be equal to (2.83 ± 0.05) × 10^16^ photons/gram. This storage capacity is only a factor five lower than the storage capacity of the commercial bulk SrAl_2_O_4_:Eu,Dy benchmark phosphor, which has a particle size in the order of 10 µm, and is a factor 20 higher than the CaS:Eu nanophosphor discussed in our previous work [32]. This result is especially surprising because a reduction of the particle size is usually detrimental to the phosphor’s afterglow performance and nano-sized phosphors generally perform worse than their bulk counterparts [23,36,37]. 

**Figure 4:**
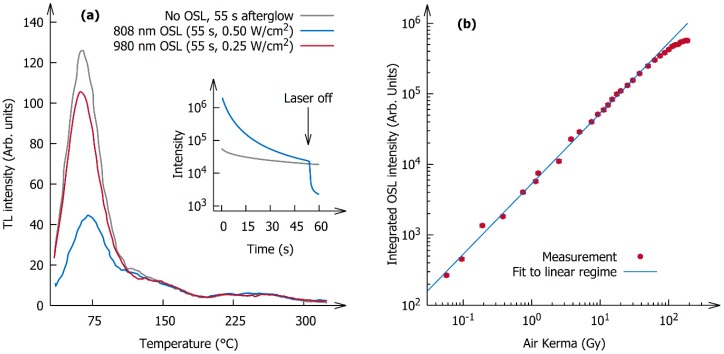
(**a**) Afterglow and thermoluminescence (TL) curves with and without optical stimulation; (**b**) dose dependence of the integrated optically stimulated luminescence (OSL) intensity (stimulated at 808 nm, 0.50 W/cm^2^).

As an alternative to thermal stimulation, optical stimulation can be used to empty the filled traps, thereby preventing degradation due to oleate oxidation. Exposing the phosphor to infrared light after irradiation with X-rays results in bright OSL. The inset in Figure 4a shows the difference between an afterglow curve with and without stimulation with 808 nm light (0.50 W/cm^2^) following 600 s (~150 Gy) of X-ray irradiation. After switching off the laser, the emission intensity of the optically stimulated sample dropped well below the afterglow intensity of the non-stimulated sample, indicating that the optical stimulation empties the shallow traps responsible for the afterglow. 

This was confirmed by combined OSL and TL experiments. Figure 4a shows the TL glow curves after 600 s of X-ray irradiation (~150 Gy) followed by 55 s of afterglow on the one hand, or after optical stimulation with infrared light for 55 s on the other hand, in order to keep the same time between the end of the excitation and the start of the TL experiment. A comparison of the curves showed that only the low temperature peak located at 65 °C is subject to optical stimulation, whereas the higher temperature glow peaks cannot be emptied optically by light with a wavelength of 808 nm or 980 nm. 

In order for the phosphor to be applicable as an optically stimulable dosimeter, the radiation dose response of the OSL should be linear or nonlinearity should be corrected for. This dose response was measured on a transparent polymer layer with a low phosphor loading of 1.6 mg/cm^2^ and using an 808 nm laser (0.50 W/cm^2^). Optical stimulation immediately followed the X-ray irradiation and the sample was thermally cleaned (heated up to 120 °C) after every measurement to ensure that there was no buildup of trapped charges between successive measurements. The OSL intensity increased linearly for doses up to 30 Gy but leveled off at higher doses due to saturation effects. This dose range falls exactly in the radiation therapy dose regime, enabling applications for external beam dosimetry and brachytherapy dosimetry where fractions of 2–20 Gy are common practice. The dose range demonstrated here for doses as low as 50 mGy could be extended further to even lower doses by increasing the concentration of nanoparticles in the dosimeter sample, thereby also enabling applications in radiation protection dosimetry. One can also envisage in vivo applications of direct cellular dosimetry by measuring light output of individual nanoparticles taken up by cells receiving ionizing radiation, opening up applications in nuclear medicine dosimetry and fundamental studies of cellular response to ionizing radiation.

Commercially available OSL dosimeters based on Al_2_O_3_:C (Landauer, Inc.; Glenwood, IL, USA) are characterized by near-tissue equivalence, a relatively simple trap structure, and robust storage properties, but exhibit supralinearity of the OSL response above 3 Gy [38,39]. Nanophosphors such as those described here may prove advantageous for situations that benefit from their high mass energy absorption coefficient relative to tissue, the NIR-stimulated luminescence, and an extended linear range. For instance, nanophosphor-loaded transparent plastics could be used for high-resolution or in situ dosimetry following high single-dose radiotherapy. 

While the *Z_eff_* of the nanophosphors themselves is high, the loading level of a host matrix can be adjusted to achieve overall near-tissue equivalence depending on the beam quality. For instance, the *Z_eff_* of the nanophosphor-loaded polymer described here varies from ~8.5 at 10 keV to ~4.0–4.5 in the range of 1–10 MeV photon beams. For a 50 kVp photon spectrum (representative of electronic brachytherapy or intra-operative radiotherapy with 50 kV X-ray sources), the mean *Z_eff_* is ~7.2 (compared to, for example, breast tissue with a mean *Z_eff_* of ~4.2 under the same conditions). The energy dependence of a potential dosimeter built using the nanophosphors described here, however, can be tuned and the dosimeter can be made tissue-equivalent by homogeneously distributing the nanophosphor in a low atomic number transparent plastic or liquid, as in Bekerat et al. [40], where the same principle was used to create energy-independent radiochromic films. The clinical utility of this nanomaterial will be investigated in future work.

**Figure 5:**
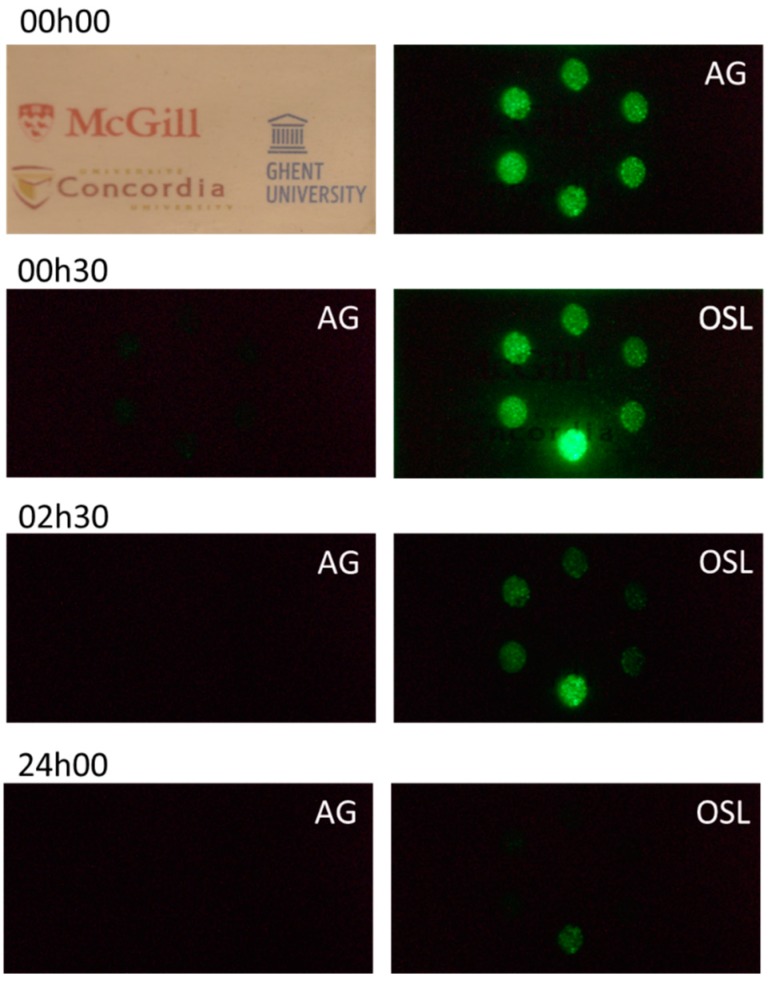
Proof of concept for dosimetric X-ray imaging. A PDMS polymer layer with 1.6 mg/cm^2^ phosphor loading (top left) was irradiated by X-rays (~150 Gy) through a mask. Afterglow after ending the X-ray irradiation (top right). Below, the remaining afterglow and OSL (808 nm, 0.50 W/cm^2^), before and during infrared irradiation, respectively, is shown for 30 min, 2.5 h, and 24 h after the X-ray exposure.

The phosphor can also be used for imaging applications. The camera image in Figure 5 (top left, under daylight) demonstrates the transparency of the PDMS layer, having a phosphor loading of 1.6 mg/cm^2^. The sample was then exposed to X-rays (corresponding to a dose of ~150 Gy) through a patterned mask, containing six circular openings, leading to a dot pattern of exposure to the X-rays. After removal of the mask, the bright afterglow (Figure 5, top right) clearly shows the parts exposed to the X-rays. When the initially bright afterglow had faded (Figure 3), OSL could be used to visualize the X-ray irradiated regions. The subsequent pictures were made just before and during the consecutive infrared irradiations at 30 min, 150 min, and 24 h after the X-ray irradiation, without re-excitation of the phosphor between successive read-out steps. The decrease of the intensity between successive pictures was mainly a consequence of previous read-outs and not due to fading. Only the dot at the bottom of the pattern was in the center of the defocused infrared laser spot (spot size of approximately 1 cm^2^). The stimulated emission from the other dots was attributed to scattered infrared light in the polymer layer and by the underlying white sheet of paper. This experiment shows that the pattern can still be visualized after multiple OSL stimulations, up to 24 h after irradiation, even with intermediate read-outs. This is indicative of both the large trapping capacity, as well as of the stability of trapped charges in the nanophosphor. This feature also enables the use of imaging (and dosimetry) systems that can be read out non-destructively, multiple times.

## 4. Conclusions

In conclusion, the Na(Gd,Lu)F_4_:Tb^3+^ phosphor shows an afterglow that is visible up to 10 h after irradiation, even when embedded at a low concentration in a polymer layer. The storage capacity was determined at (2.83 ± 0.05) × 10^16^ photons per gram. This shows that it can compete with the commercially available phosphors concerning performance, while it stands out because of the small particle size and solution processability. The phosphor can be read out, both thermally and optically, up to 24 h after excitation. Optical stimulation with infrared radiation offers a way to read out the phosphor without heating the nanoparticles, thereby avoiding the stability issues with the oleate capping. The radiation dose response is linear over three orders of magnitude up to 30 Gy.
